# The **I*N*CA** trial (**I**mpact of ***N****OD2* genotype-guided antibiotic prevention on survival in patients with liver **C**irrhosis and **A**scites): study protocol for a randomized controlled trial

**DOI:** 10.1186/s13063-015-0594-4

**Published:** 2015-03-08

**Authors:** Markus Casper, Martin Mengel, Christine Fuhrmann, Eva Herrmann, Beate Appenrodt, Peter Schiedermaier, Matthias Reichert, Tony Bruns, Cornelius Engelmann, Frank Grünhage, Frank Lammert, and the INCA trial group

**Affiliations:** Department of Medicine II, Saarland University Medical Center, Kirrberger Straße 100, 66421 Homburg, Germany; Study Center Bonn, Institute of Clinical Chemistry and Clinical Pharmacology, University Hospital Bonn, Sigmund-Freud-Straße 25, 53125 Bonn, Germany; Institute for Biostatistics and Mathematical Modelling, Goethe University Hospital, Theodor-Stern-Kai 7, 60590 Frankfurt am Main, Germany; Department of Medicine, Nardini Hospital, Kaiserstraße 14, 66482 Zweibrücken, Germany; Department of Medicine IV, University Hospital Jena, Bachstraße 18, 07743 Jena, Germany; Department of Medicine II, University Hospital Leipzig, Liebigstraße 18, 04103 Leipzig, Germany

**Keywords:** Genetic testing, Liver cirrhosis, Portal hypertension, Primary prophylaxis, Spontaneous bacterial peritonitis

## Abstract

**Background:**

Patients with liver cirrhosis have a highly elevated risk of developing bacterial infections that significantly decrease survival rates. One of the most relevant infections is spontaneous bacterial peritonitis (SBP). Recently, *NOD2* germline variants were found to be potential predictors of the development of infectious complications and mortality in patients with cirrhosis. The aim of the I*N*CA (Impact of *NOD2* genotype-guided antibiotic prevention on survival in patients with liver Cirrhosis and Ascites) trial is to investigate whether survival of this genetically defined high-risk group of patients with cirrhosis defined by the presence of *NOD2* variants is improved by primary antibiotic prophylaxis of SBP.

**Methods/Design:**

The I*N*CA trial is a double-blind, placebo-controlled clinical trial with two parallel treatment arms (arm 1: norfloxacin 400 mg once daily; arm 2: placebo once daily; 12-month treatment and observational period). Balanced randomization of 186 eligible patients with stratification for the protein content of the ascites (<15 versus ≥15 g/L) and the study site is planned. In this multicenter national study, patients are recruited in at least 13 centers throughout Germany. The key inclusion criterion is the presence of a *NOD2* risk variant in patients with decompensated liver cirrhosis. The most important exclusion criteria are current SBP or previous history of SBP and any long-term antibiotic prophylaxis. The primary endpoint is overall survival after 12 months of treatment. Secondary objectives are to evaluate whether the frequencies of SBP and other clinically relevant infections necessitating antibiotic treatment, as well as the total duration of unplanned hospitalization due to cirrhosis, differ in both study arms. Recruitment started in February 2014.

**Discussion:**

Preventive strategies are required to avoid life-threatening infections in patients with liver cirrhosis, but unselected use of antibiotics can trigger resistant bacteria and worsen outcome. Thus, individualized approaches that direct intervention only to patients with the highest risk are urgently needed. This trial meets this need by suggesting stratified prevention based on genetic risk assessment. To our knowledge, the I*N*CA trial is first in the field of hepatology aimed at rapidly transferring and validating information on individual genetic risk into clinical decision algorithms.

**Trial registrations:**

German Clinical Trials Register DRKS00005616. Registered 22 January 2014.

EU Clinical Trials Register EudraCT 2013-001626-26. Registered 26 January 2015.

## Background

Cirrhosis is the final common pathway of chronic liver diseases [1]. The alcoholic and non-alcoholic fatty liver diseases, as well as chronic viral hepatitis, are the most important causes of cirrhosis. The increasing liver disease rates, with more than 800,000 annual deaths worldwide due to complications of cirrhosis [2], demonstrate the high socioeconomic impact of liver diseases and the need for improved patient care and disease management.

Patients with cirrhosis are approximately ten times more likely to develop bacterial infections than healthy persons [3]. Spontaneous bacterial peritonitis (SBP) and urinary tract infections are observed most frequently [4]. The occurrence of bacterial infections is also highly relevant for the patients’ prognosis, with the overall median mortality of infected patients with cirrhosis quadrupling to more than 60% at 12 months [3]. The prevalence of SBP is 10% in hospitalized patients and 1.5% to 3.5% in outpatients with cirrhosis [5,6]. Although antibiotic therapy reduces the acute mortality of hospital inpatients with SBP, there is a very high probability of recurrence and death (up to 70%) within 1 year after an episode of SBP [7-9].

Long-term antibiotic prophylaxis has been shown in randomized controlled trials to significantly reduce the risk of SBP recurrence [7], and it is thus advised for all patients who have survived an index SBP [10]. The best data exist for fluoroquinolones, especially for norfloxacin [7,11]. Primary prevention of SBP with antibiotics to avoid a first episode is currently not uniformly recommended for patients with ascites, because the existing data are conflicting and the intervention might promote the development of multidrug-resistant bacteria [12-16]. Thus, long-term antibiotic prophylaxis should be administered only to subgroups with the highest risk [10]. In one randomized controlled trial, Fernández and co-workers showed that primary prophylaxis with norfloxacin in a clinically defined high-risk population (protein level of the ascites below 15 g/L and advanced liver failure or impaired renal function) significantly reduced the risk for a first episode of SBP and short-time survival [17].

Recently, the *NOD2* (nucleotide-binding oligomerization domain containing 2; HGVS name: NC_000016.10) germline variants p.R702W, p.G908R and c.3020insC were found to be predictors of the development of infectious complications and mortality in patients with cirrhosis when carriers of the variants died significantly earlier and significantly more often experienced SBP [18]. Bruns *et al*. [19] validated the association between *NOD2* variants and culture-positive SBP. Their study indicated that patients carrying *NOD2* variants presented more frequently with variceal bleeding and hepatocellular carcinoma. Thus, it is the aim of the I*N*CA (Impact of *NOD2* genotype-guided antibiotic prevention on survival in patients with liver Cirrhosis and Ascites) trial to investigate whether survival of a genetically defined high-risk group of cirrhotic patients characterized by the presence of *NOD2* genetic variation is improved by primary antibiotic prophylaxis of SBP.

## Methods/Design

### Trial design and ethical considerations

**Figure 1:**
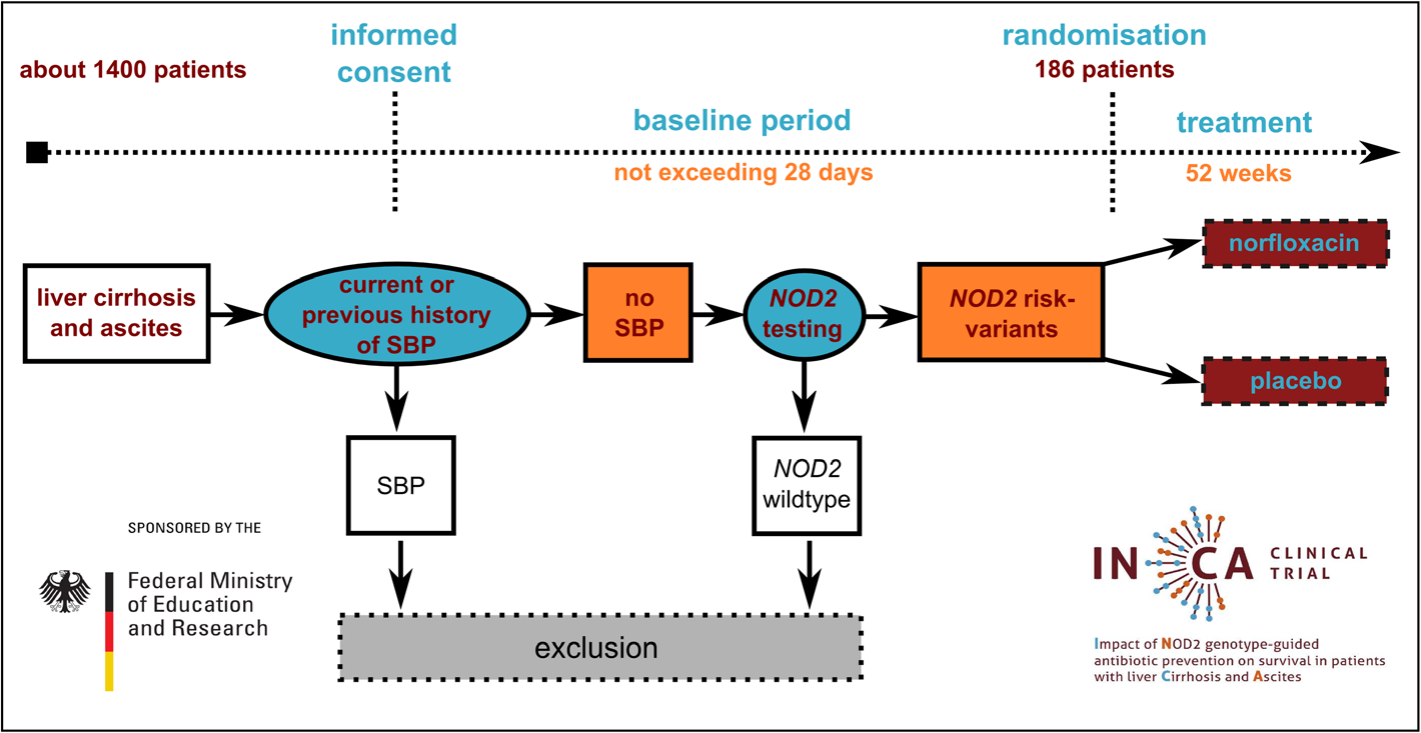
**Study flowchart.** Overall, 186 patients meeting the inclusion criteria and no exclusion criteria are randomly assigned to two treatment arms. Especially, present SBP has to be ruled out, and patients have to be verified to carry at least one of the three common *NOD2* variants. It is expected that about 1,400 patients need to be evaluated to finally randomize the calculated 186 patients. SBP, Spontaneous bacterial peritonitis.

The trial has been designed to assess the effect of antibiotic primary prophylaxis on survival in carriers of *NOD2* risk variants without SBP. Figure 1 summarizes the design of the study. The I*N*CA trial is a double-blind, placebo-controlled clinical trial with two parallel treatment arms. It is planned to randomly allocate 186 eligible patients to one of these arms (1:1 ratio). Balanced randomization, stratified for the protein content of the ascites (<15 g/L or ≥15 g/L, given that a low protein content is an established risk factor for SBP) and the study center, will be performed as centralized, computer-based block randomization using separate randomization lists for each stratum. Only the central and independent pharmacy (Heidelberg University Hospital) performing the randomization procedure, and neither the investigators nor the patients, will be aware of the allocation sequence or the block size used. Blinding of participants and study personnel responsible for treatment and outcome assessment is ensured by identical encapsulation of placebo and active substance that makes both indistinguishable, as well as by the use of identical blisters and folding boxes. The unique patient randomization number labeled on the boxes makes the medication patient-specific. The confidential block size ensures randomization concealment after emergency un-blinding because of safety reasons or after unblinding due to SBP. Access to emergency envelopes is regulated at all sites.

The patients allocated to arm 1 receive 400-mg norfloxacin capsules (Norfloxacina ABC; ABC International Pharma, Ivrea, Italy) once daily. In arm 2, the patients receive an identically looking capsule containing no active ingredient (placebo) once daily. The treatment and observational periods are 12 months for both groups. Treatment will not differ between arms.

The study is approved by the leading ethics committee (Ethikkommission der Ärztekammer des Saarlandes; reference number 71/13) and the German Federal Institute for Drugs and Medical Devices (Bundesinstitut für Arzneimittel und Medizinprodukte, BfArM; reference number 4039362). The participating centers’ eligibilities were evaluated by the responsible local ethics committees (University Hospital Bonn: Ethikkommission an der Medizinischen Fakultät der Rheinischen Friedrich-Wilhelms-Universität Bonn; University Hospital Essen: Ethik-Kommission der Medizinischen Fakultät der Universität Duisburg-Essen; University Hospital Frankfurt: Ethik-Kommission des Fachbereichs Medizin der Johann Wolfgang Goethe-Universität Frankfurt; University Hospital Halle: Ethik-Kommission der Medizinischen Fakultät der Martin-Luther-Universität Halle-Wittenberg; University Hospital Hamburg-Eppendorf: Ethik-Kommission der Ärztekammer Hamburg; University Hospital Heidelberg: Ethikkommission der Medizinischen Fakultät Heidelberg; University Hospital Jena: Ethik-Kommission der Medizinischen Fakultät der Friedrich-Schiller-Universität Jena; Westpfalz-Hospital Kaiserslautern and University Hospital Mainz: Ethik-Kommission der Landesärztekammer Rheinland-Pfalz; University Hospital Leipzig: Ethik-Kommission an der Medizinischen Fakultät der Universität Leipzig; University Hospital Mannheim: Medizinische Ethikkommission II der Medizinischen Fakultät; University Hospital Ulm: Ethik-Kommission der Universität Ulm). The I*N*CA trial is registered in the EU Clinical Trials Register (EudraCT 2013-001626-26) and will be conducted in accordance with the Declaration of Helsinki in its latest version, the guidelines of the International Conference on Harmonization of Good Clinical Practice and the applicable German law. In order to ensure patient safety, an external data and safety monitoring board (DSMB) consisting of at least three independent members with a high level of expertise in the conduct of clinical trials, as well as in the fields of hepatology, pharmacology and statistics, will receive unblinded safety and outcome data at defined time points (after inclusion of 10, 60 and 100 patients).

### Participants

In general, all patients with liver cirrhosis and ascites should be screened for the I*N*CA trial. Importantly, only patients verified to carry at least one of the three common *NOD2* risk variants (p.R702W, p.G908R or c3020insC) can ultimately participate in the trial. The most important exclusion criteria are a present SBP or a previous history of such, as well as long-term antibiotic treatment, irrespective of the indication. Table 1 provides a list of the inclusion and exclusion criteria. In this multicenter national study, patients will be recruited at a minimum of 13 participating referral centers. These centers have specific clinical expertise in treating patients with advanced liver diseases, and ten of them are centers for liver transplantation. These centers were also selected because of their experience in conducting randomized, controlled trials, their specialized outpatient structure and their collaboration with local primary and secondary care hospitals. The estimated recruitment period is 24 months, and recruitment started in February 2014.

**Table 1:** **Inclusion and exclusion criteria** ^**a**^

**Inclusion criteria**	**Exclusion criteria**
Age ≥18 years.	Age <18 years.
Written informed consent to participate in the clinical trial and written informed consent for genetic testing.	Absent written informed consent to participate in the clinical trial or for genetic testing.
Patients have to be able to understand and follow instructions and to be willing to attend all study visits (compliance).	Patients unable to understand the meaning of the clinical trial and the consequences of study participation.
Presence or history of ascites in case of advanced liver disease compatible with cirrhosis (liver biopsy not required).	Patients unable to understand or follow instructions or not willing to attend all study visits.
Diagnostic paracentesis to exclude SBP within 10 days before the baseline visit. Patients who cannot undergo paracentesis because of small amounts of ascites can only be included in the trial if SBP is unlikely, taking into account clinical and blood test indicators.	Simultaneous participation in another clinical trial (study medication has to be stopped for almost 30 days before the baseline visit).
Positive genotyping result for at least one of the *NOD2* risk variants p.R702W, p.G908R or c.3020insC.	Persistent drug abuse (alcohol abuse may be tolerated in the setting of adequate compliance).
Pregnancy is to be excluded by a pregnancy test (beta-hCG blood test or urine test) in women with childbearing potential who have not undergone surgical contraceptive methods or hysterectomy. These patients have to use effective contraceptive methods.	Pregnancy, planned pregnancy or breastfeeding patients.
	Patients without a history of ascites.
	SBP diagnosed by the index paracentesis within 10 days before baseline.
	Previous history of SBP. (When this is uncertain, absence of a secondary antibiotic prophylaxis may be used as an alternative criterion to exclude SBP.)
	Long-term antibiotic prophylaxis, irrespective of the indication. Long-term treatment is to be completed at least 28 days before randomization.
	Contraindications against norfloxacin or placebo such as the following:
	• Intolerance to norfloxacin, to substances with related chemical structure or to other components of norfloxacin or placebo.
	• Patients with acquired long QT syndrome or other nonmodifiable risk factors causing a persisting corrected QT prolongation (corrected according to Bazett’s formula: >470 ms for men and >480 ms for women).
	• Patients with galactose intolerance, lactamase deficiency or glucose and/or galactose malabsorption.
	• Patients with myasthenia gravis.
	• Patients with tendinitis or tendon rupture linked to fluoroquinolone intake.
	Patients with a life expectancy of less than 12 months due to hepatocellular cancer, other malignant diseases or another severe comorbidity.
	Patients with HIV infection with a CDC classification clinical stage C or laboratory stage 3.

^a^CDC, Centers for Disease Control and Prevention; hGC, Human chorionic gonadotropin; *NOD2*, nucleotide-binding oligomerization domain containing 2; SBP, Spontaneous bacterial peritonitis.

### Objectives and endpoints

The I*N*CA trial design has been chosen to determine whether primary antibiotic prophylaxis with norfloxacin improves overall survival in a high-risk population of patients with liver cirrhosis and ascites defined by the *NOD2* genotype. The secondary aims are to evaluate if the frequencies of SBP and other clinically relevant infections necessitating antibiotic treatment (for example, urinary tract infection, pneumonia, sepsis, bacteremia), as well as the total duration of unplanned hospitalization due to cirrhosis, differ between study arms. Table 2 summarizes the study endpoints. In addition, safety aspects, including the impact of norfloxacin on the intestinal microbiome, will be addressed.

**Table 2:** **Study endpoints**

**Primary endpoint**	**Secondary endpoints**
Overall survival after 12 months	Spontaneous bacterial peritonitis within 12 months
	Other clinically significant infections (for example, urinary tract infections, pneumonia, sepsis, bacteremia) requiring antimicrobial treatment within 12 months
	Duration of unscheduled cirrhosis-associated hospitalization within 12 months

### Frequency and scope of study visits and interventions

All patients who have already developed ascites should be screened for trial participation. Particularly, SBP has to be excluded, and ascitic protein content has to be determined by a clinically indicated index paracentesis (maximally 10 days prior to baseline visit). Patients who cannot undergo paracentesis because of small amounts of ascites can be included in the trial only if SBP is unlikely, taking into account clinical and blood test indicators. Hereafter potentially eligible patients must be informed about the study and genetic testing by an investigator with the use of a specific information sheet. Informed consent to study participation and genetic testing is mandatory for a further evaluation of patients, and especially *NOD2* genetic testing, using patients’ blood specimens. At the baseline visit (within 28 days before randomization) and during treatment within the trial, only noninvasive or minimally invasive interventions are scheduled (Table 3). Patients who fulfill all inclusion criteria but no exclusion criteria proceed to randomization. Treatment with the study medication must be initiated within 7 days after randomization. Adverse reactions to the study medication are more likely to occur early after treatment initiation, so that closely scheduled visits are implemented for the first 4 weeks. Thereafter, and for the remainder of the trial, the study visits are less frequent (Table 4) and, at predefined time points only, telephone interviews are scheduled to record any (serious) adverse event (AE) as well as primary and secondary endpoint information. Patients are regularly treated within the trial for 12 months. Patients who must definitely stop the study medication for any reason (for example, SBP,prolongation of QTc above 500ms [QTc; QT interval corrected for heart frequency using Bazett’s formula]) will complete the trial without taking the trial medication and attend the regular visits. Patients with SBP during the trial will be unblinded. Patients who undergo liver transplantation or who revoke their consent to participate are censored for the analysis with the date of withdrawal.

**Table 3:** **Study-specific actions** ^**a**^

**Action**	**Baseline period**	**Study visits**	**Telephone visits**
Informed consent	X		
Checking inclusion and exclusion criteria	X		
Demographics	X		
Medical history (with focus on liver disease)	X		
*NOD2* genetic testing	X		
Concomitant diseases	X	X	X
Concomitant medications	X	X	X
MELD and Child-Pugh-scores	X	X	
Clinical assessment and vital signs	X	X	
12-lead ECG	X	X	
Blood tests (safety parameters)	X	X	
Recording of adverse events		X	X
Distribution and return of study medication		X	
Collection of ascites samples (clinically indicated puncture)	X	X	
Collection of stool samples		X	

^a^ECG, Electrocardiography; MELD, Model For End-Stage Liver Disease.

**Table 4:** **Visits and time points**

**Baseline period**	**Within 28 days before randomization**
Visit 1	Day 0 (up to 7 days after randomization)
Visit 2	Day 7 (±2 days)
Telephone visits 1 to 9	Weeks 4, 8, 16, 20, 28, 32, 40, 44 and 48 (±7 days)
Visits 3 to 5	Weeks 12, 24 and 36 (±7 days)
Visit 6	Week 52 (±7 days) or shortly after close-out

Patients are allowed any additional, necessary treatment, which is at the discretion of the physician in charge. However, to avoid treatment bias, any long-term treatment with antibiotics is prohibited, whereas temporary antibiotics for treatment of acute infections are permitted. Study medication can be paused for a maximum of 7 days.

In case of AEs, the investigators at each trial center judge the severity and causality of the AE and decide on an individual basis to continue, pause or terminate the study drug. The compliance of study participants is determined by the ratio of pills actually taken (pills delivered minus pills returned) and pills expected to be taken.

Because treatment with fluoroquinolones rarely causes QT interval prolongation, electrocardiography (ECG) with determination of the QTc is mandatory for safety reasons at baseline, to visit 1 before first intake of study medication as well as to study visits 2 through 6 during trial participation. The actions to be taken in case of marked prolongation of the QTc (ECG controls, modification of concomitant medication, pausing or termination of the study drug) are in line with published guidelines and recommendations [20,21].

At the baseline visit, an ascites sample derived from the index paracentesis is collected and stored. In case of recurrent ascites and suspicion of SBP, a diagnostic paracentesis must be performed (according to standard of care) to diagnose or exclude SBP. In this case, ascites samples are collected. Moreover, stool samples are collected regularly throughout the study (visits 1 through 6) for additional analyses to evaluate the effects of long-term treatment with antibiotics on intestinal microbiome composition (sequence-based analyses investigating absolute and relative abundance as well as diversity of microorganisms; sequence- and culture-based resistance analyses).

### Statistical analyses

#### Primary statistical aim

The confirmatory part of the statistical analysis is the assessment of treatment efficacy by testing the null hypothesis (H0), “The survival of patients treated with norfloxacin is equal to the survival of patients treated with placebo”, against the alternative hypothesis (H1), “The survival of patients treated with norfloxacin is better than the survival of patients treated with placebo”, by using a one-sided log-rank test with a significance level α = 5%. Antibiotic primary and secondary SBP prophylaxis with norfloxacin showed no negative effects on survival in previous trials and meta-analyses [7,10,12,13,22]. Moreover, there is no evidence for a preponderance of deleterious treatment effects associated with norfloxacin in this patient group, which justifies a one-sided test. All patients who receive at least one dose of norfloxacin or placebo are included in the analysis as an intention-to-treat approach. Incomplete information is accounted for by censoring.

#### Secondary statistical aims

As an exploratory analysis, and in order to identify further predictor variables for survival, a multivariate Cox regression method is used. It incorporates baseline variables and the occurrence of infectious complications that require antibiotics as time-dependent variables. Tests are two-sided with a significance level of α = 5%. To avoid overfitting, we apply the rule of thumb and include at most enough independent variables that ten or more events per independent variable are still observed.

#### Safety

Safety parameters are assessed by competing risk analysis. In addition, descriptive statistics on safety parameters are added, using two-sided tests at a significance level of 5%.

#### Sample size calculation

To identify a difference of 20% survival rate after 12 months (60% versus 40%, one-sided log-rank test, α = 0.05, 1 − β = 0.8), and to account for a 17% loss due to dropout during treatment, 186 patients carrying *NOD2* variants have to be included in the trial. Sample size calculation is based on the previous observation that patients carrying at least one *NOD2* variant have a deleterious outcome with a survival of only 40% within 12 months, as compared to 73% of patients with wild-type genotypes at all three *NOD2* loci [18], and an increase in survival rate from 48% to 60% after 12 months in a recent randomized controlled trial in which SBP primary prophylaxis was investigated without taking *NOD2* genotypes into account [17]. In studies in which norfloxacin was administered for 12 months or longer to patients with liver cirrhosis and ascites, non-compliance rates ranged from 5% to 8% [17,23]. Although loss to follow-up has been inconsistently reported, the two landmark studies on secondary SBP prophylaxis had rates of up to 9% for loss to follow-up. As a conservative estimate, we calculated our projected sample size with the reported maximum of 17% loss to follow-up and non-compliance. Among all patients considered for the I*N*CA trial, only 25% carry at least one *NOD2* risk allele [18]. Moreover, we expect that 25% of eligible patients to drop out because of lack of informed consent, and a maximum of 10% of the total number of patients with ascites are expected to present with SBP, leading to exclusion. Thus, a total of 1,380 patients with cirrhosis and ascites initially need to be evaluated for the study. Patients evaluated but not included are documented and reported according to the CONSORT statement (http://www.consort-statement.org/).

## Discussion

The impaired intestinal mucosal barrier with subsequent bacterial translocation is considered to represent one of the key pathophysiological mechanisms leading to SBP in patients with cirrhosis [24,25]. In 2001, variants of the *NOD2* gene were associated with impaired mucosal barrier function in Crohn’s disease [26]. Because *NOD2* is involved in the intestinal recognition of bacteria, insufficient activation of nuclear factor κB and recruitment of autophagy-related proteins in carriers of *NOD2* risk variants might result in deficient destruction of bacteria and promote their translocation from the intestine [27]. In line with this, the *NOD2* germline variants p.R702W, p.G908R and c.3020insC were found to be predictors of the development of infectious complications and mortality in patients with cirrhosis [18,19]. It has also been reported that the *NOD2* variants are associated with reduced survival in sepsis [28].

In 2007, a randomized controlled trial in which a clinically defined high-risk group of patients with cirrhosis and ascites was investigated showed that primary prophylaxis with norfloxacin improved the 3-month probability of survival (94% versus 63%; *P* =0.03) [17]. However, according to a recent Cochrane review [14], all previous studies (538 patients) on primary SBP prophylaxis were underpowered to assess survival over a 12-month period. Thus, further trials are needed to substantiate prevention strategies. Because broad-spectrum antibiotic prophylaxis might be hampered by the selection of resistant bacteria [29], long-term antibiotics should be administered only to the subgroups with the highest risk, which has yet to be defined [10,30].

The I*N*CA trial thus evaluates the effect of antibiotic primary prophylaxis on survival in a genetically defined high-risk group. Because no gold standard for the management of patients with cirrhosis with ascites, but without SBP, has been established and current consensus guidelines [10] have not implemented general recommendations for antibiotic primary prophylaxis in these patients, randomization to placebo and surveillance of patients with wild-type *NOD2* is admissible and ethical. Data suggesting beneficial effects of antibiotic prophylaxis in patients with cirrhosis with low ascitic protein content (<15 g/L) is limited, and survival analyses are conflicting [14]. Hence, until more reliable data are available, randomization to placebo for these patients has been considered ethical. In cases of obvious disadvantages for the patients in the low ascitic protein content stratum treated with placebo, the DSMB will propose appropriate measures so that there are no uncontrolled risks for participating patients.

Norfloxacin, a poorly absorbed antibiotic with activity predominantly against Gram-negative bacteria, has been widely studied in patients with liver cirrhosis and is documented to be safe in these patients. Alternative drugs include ciprofloxacin or co-trimoxazole, but evidence for SBP prophylaxis is not as robust as that of norfloxacin [10]. The fully non-absorbable antibiotic rifaximin represents a promising alternative, but it has not yet been assessed in randomized controlled trials for prevention of SBP [31-33]. The norfloxacin dose of 400 mg per day is chosen because it was used successfully in previous studies for the primary prophylaxis of SBP in patients with low ascitic protein content [17,23], and this is the standard dose for secondary prophylaxis of SBP [10,30].

The primary endpoint of the I*N*CA trial is the overall survival over a period of 12 months. This endpoint has been chosen to assess the benefit of the intervention for patients in relation to current average waiting times for liver transplantation [34]. The occurrence of SBP has been selected as a secondary endpoint because the hypothesis underlying antibiotic prophylaxis refers to the impaired mucosal barrier in the intestine causing intra-abdominal infections such as SBP [24,25]. In case of SBP during study participation, microbiological analyses may help to calculate the frequency of quinolone-resistant SBP. Although it has been shown that these infections respond to the recommended antibiotics in most cases [14], they may confer a specific risk for patients receiving norfloxacin. Specimens taken during the study (ascites, stool) may enable us to clarify whether a potentially increased frequency of infections with resistant bacteria is due to intestinal selection or selective translocation of quinolone-resistant bacteria or whether it is associated with specific changes or “enterotypes” of the intestinal microbiome [35,36]. The occurrence of any clinically significant infection other than SBP has been chosen as a secondary endpoint because the spread of bacteria across the intestinal mucosal barrier could promote other infectious complications also influencing the patients’ outcome. An excess of infections requiring antibiotic treatment in one of the treatment arms provides information on whether antibiotic prophylaxis promotes or avoids potentially life-threatening infections at other sites. Finally, the secondary endpoint of hospitalization allows us to assess health care costs and quality of life.

For safety reasons, special attention is paid to the occurrence of AEs throughout the I*N*CA trial. Owing to the high *a priori* risk of trial participants, we expect a large number of AEs and serious AEs. Moreover, we predict a mortality of up to 60% in our cohort. To avoid excess mortality in one of the study arms, the progress of the I*N*CA trial is supervised by a DSMB.

Individualized diagnosis and treatment approaches are key themes for future research directives and may substantially change health care for individual patients. Up to 50% of patients awaiting liver transplantation die as a result of infectious complications [37]. Although preventive strategies are required to avoid life-threatening infections in these patients, a broad and unselected use of antibiotics can also trigger resistant bacteria and worsen outcome. Thus, a better selection of patients and personalized approaches that direct interventions only to patients with the highest risk are urgently needed [30]. The I*N*CA trial meets this need by suggesting stratified prevention based on risk assessment. The I*N*CA trial is the first in the field of hepatology with an aim to rapidly transfer and validate information on individual genetic risk into clinical decision algorithms.

## Trial status

The trial started recruitment in February 2014. Recruitment may be finished in February 2016.

## Abbreviations

AE: Adverse event
DSMB: Data and safety monitoring board
ECG: Electrocardiography
*NOD2*: Nucleotide-binding oligomerization domain containing 2 gene
QTc: QT interval corrected for heart frequency
SBP: Spontaneous bacterial peritonitis
